# Chiral Hybrid Perovskite Single‐Crystal Nanowire Arrays for High‐Performance Circularly Polarized Light Detection

**DOI:** 10.1002/advs.202102065

**Published:** 2021-09-24

**Authors:** Zhen Liu, Chunhuan Zhang, Xiaolong Liu, Ang Ren, Zhonghao Zhou, Chan Qiao, Yuwei Guan, Yuqing Fan, Fengqin Hu, Yong Sheng Zhao

**Affiliations:** ^1^ College of Chemistry Beijing Normal University Beijing 100875 China; ^2^ Key Laboratory of Photochemistry Institute of Chemistry Chinese Academy of Sciences Beijing 100190 China; ^3^ University of Chinese Academy of Sciences Beijing 100049 China

**Keywords:** chiral hybrid perovskites, circularly polarized light detection, nanowire arrays, perovskite nanowires, perovskite photodetectors

## Abstract

Circularly polarized light (CPL) detection has emerged as a key technology for various optoelectronics. Chiral hybrid perovskites (CHPs) that combine CPL‐sensitive absorption induced by chiral organic ligands and superior photoelectric properties of perovskites are promising candidates for direct CPL detection. To date, most of the CHP detectors are made up of polycrystalline thin‐film, which results in a rather limited discrimination of CPL due to the existence of redundant impurities and intrinsic defect states originating from rapid crystallization process. Here, it is developed a direct CPL detector with high photocurrent and polarization selectivity based on low‐defect CHP single‐crystal nanowire arrays. Large‐scale CHP nanowires are obtained through a micropillar template‐assisted capillary‐bridge rise approach. Thanks to the high crystallinity and ordered crystallographic alignment of these arrays, a CPL photodetector with high light on/off ratio of 1.8 × 10^4^, excellent responsivity of 1.4 A W^−1^, and an outstanding anisotropy factor of 0.24 for photocurrent has been achieved. These results would provide useful enlightenment for direct CPL detection in high‐performance chiral optoelectronics.

## Graphical abstract

An efficient circularly polarized light (CPL) detector is demonstrated with large‐scale chiral hybrid perovskite (CHP) nanowire arrays. Based on the high photocurrent and polarization selectivity of CHP single‐crystals, high‐performance CPL detectors with high light on/off ratio and excellent polarization distinguishability through arranging such single‐crystalline CHP nanowires into low‐defect arrays are achieved.

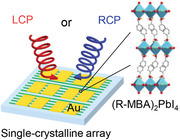

## 1 Introduction

Circularly polarized light (CPL) photodetectors have been demonstrated to be an important type of tool in a wide variety of optoelectronic fields, such as drug screening, security system, remote sensing, and quantum optics.^[^ ^1^, ^2^, ^3^, ^4^, ^5^ ^]^ Conventional CPL detectors require the combination of a non‐chiral photodetector with a quarter waveplate and a linear polarizer,^[^ ^6^, ^7^ ^]^ which hinders their miniaturization and integration for ultra‐compact optoelectronic devices. In contrast, optically active chiral materials could naturally differentiate left‐handed circularly polarized (LCP) light and right‐handed circularly polarized (RCP) light by virtue of their intrinsic chirality, offering an appealing option to construct direct CPL photodetectors. A typical example is chiral organic compounds, which have been utilized for direct CPL detection devices benefiting from the intrinsic chiral response of organic molecules.^[^ ^1^, ^8^ ^]^ However, these photodetectors generally suffer from low responsivity owing to the poor charge carrier mobility and low exciton dissociation energy of organic molecules, hence impeding their practical applications for sensitive CPL detection. Therefore, developing photoactive materials that possess both CPL‐sensitive optical absorption and efficient charge transport properties is a prerequisite for high‐performance CPL detection.

Chiral hybrid perovskites (CHPs),^[^ ^9^, ^10^ ^]^ featured with handedness sensitive absorption induced by chiral organic ligands and superior optoelectronic properties originating from inorganic frameworks,^[^ ^11^, ^12^, ^13^, ^14^, ^15^, ^16^, ^17^ ^]^ are ideal candidates for the construction of high‐performance direct CPL detector. Over the past few years, thin‐film‐based CHPs photodetectors have enabled direct detection of light polarization states with enhanced responsivity;^[^ ^18^, ^19^, ^20^, ^21^, ^22^ ^]^ however, the capability of these devices to distinguish CPL is still limited due to the introduction of impurities and intrinsic defect states during the rapid crystallization process.^[^ ^23^ ^]^ With low defect density and long carrier diffusion length stemming from the long‐range crystallographic order,^[^ ^24^, ^25^, ^26^, ^27^, ^28^ ^]^ high‐crystallinity CHP as active component affords an opportunity to detect CPL with high polarization discrimination ratio.^[^ ^29^, ^30^ ^]^ Furthermore, arranging such CHP into an ordered array would not only enhance the photocurrent by enlarging the active area, but also bring excellent uniformity and reproducibility, which are of great importance for the practical photodetection technology.^[^ ^31^, ^32^, ^33^, ^34^, ^35^, ^36^ ^]^ Therefore, the development of large‐scale CHP arrays with high crystallinity and homogeneous morphology would vastly promote the direct CPL detection applications in high‐performance chiral selective optoelectronics.

Herein, we demonstrate the realization of direct CPL detection with high photocurrent and polarization selectivity based on CHP single‐crystalline nanowire (NW) arrays. Through a micropillar template‐assisted capillary‐bridge rise approach, large‐scale CHP NWs with uniform morphology as well as controlled location and size were obtained from the precursor solution of CHP single‐crystal. With high crystallinity and ordered crystallographic alignment, these fabricated CHP single‐ crystal microstructures possess reduced charge trap density and extended carrier lifetimes, which are beneficial to facilitate the charge transport efficiency. As a result, excellent optoelectronic performance with high light on/off ratio of 1.8 × 10^4^, and the responsivity up to 1.4 A/W was demonstrated in the single‐crystal NW array‐based CPL photodetector. In particular, this CPL detector exhibits high distinguishability with an outstanding anisotropy factor of 0.24 for photocurrent, which can be attributed to the long spin lifetimes in high‐crystallinity and low‐defect single‐crystalline arrays. These results provide a useful reference for the realization of high‐performance direct CPL detection, paving the way for chiral sensing and imaging applications.

## 2 Results and Discussions

**Scheme 1:**
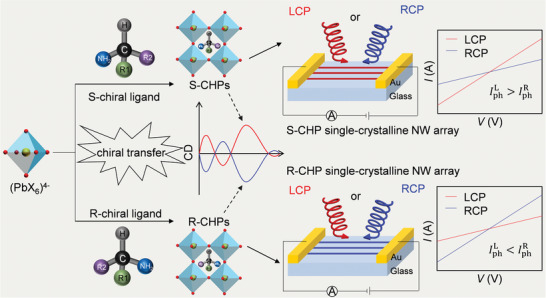
Design of CHP single‐crystalline array for achieving high‐performance direct CPL photodetection. By incorporating chiral organic ligands into the inorganic octahedral framework (PbX_6_)^4−^ of perovskites, the optically active chiral semiconductor with efficient charge transport properties can be obtained. The as‐prepared CHPs will exhibit opposite CD signals owing to the chiral transfer of ligands. Arranging such CHPs into a single‐crystalline NW array with low defect and long‐range crystallographic order will enable to improve the polarization distinguishability and charge transport efficiency, thereby a high‐performance direct CPL detector will be expected.

**Scheme** **1** illustrates the design principle of CHP single‐crystalline arrays for achieving high‐performance direct CPL photodetection. The optically active chiral semiconductor with efficient charge transport properties can be obtained by incorporating chiral organic ligands into the inorganic octahedral framework (PbX_6_)^4−^ of perovskites. These chiral ligands should have big *π* bond in the benzene ring to facilitate the coulomb interaction between chiral amines and (PbI_6_)^4−^ matrix for the realization of enhanced CPL‐sensitive absorption.^[^ ^18^ ^]^ The as‐prepared CHPs would exhibit opposite circular dichroism (CD) signals owing to the chiral transfer of ligands, which is the prerequisite for the construction of direct CPL detectors. Arranging such chiral hybrid units into a low defect and long‐range crystallographic ordered single‐crystalline form will enable to improve the polarization distinguishability and charge transport efficiency, thereby a high‐performance direct CPL detector would be expected.

**Figure 1:**
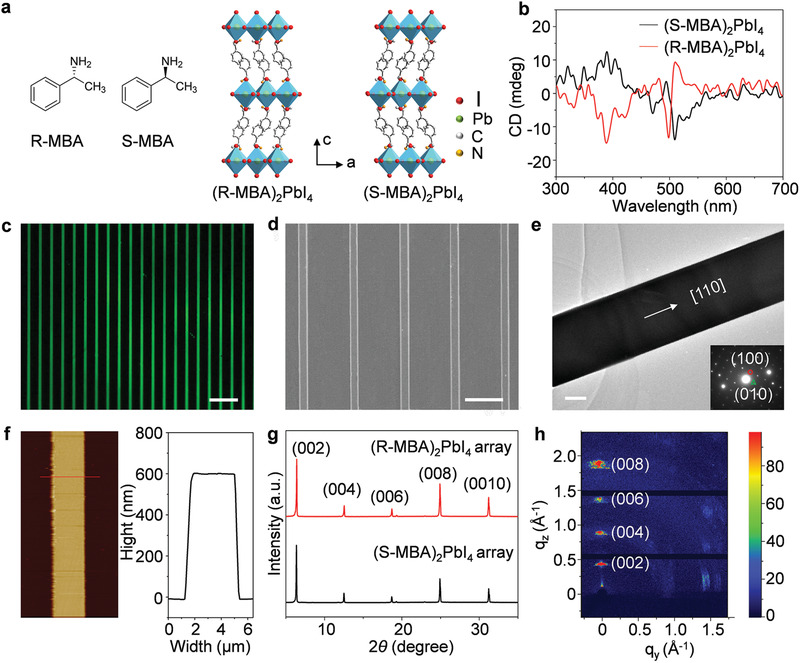
Fabrication and crystal structure of the CHP single‐crystalline arrays. a) Left: molecular structures of R‐MBA and S‐MBA chiral ligands. Right: crystal structures of (R‐ and S‐MBA)_2_PbI_4_ CHPs. b) CD spectra of (S‐MBA)_2_PbI_4_ and (R‐MBA)_2_PbI_4_ films. Strong CD signals are observed at almost the same wavelengths (at 331, 389, 481, 497, and 510 nm) but with oppositely signed values. c) Fluorescence microscopy image of a large‐area (S‐MBA)_2_PbI_4_ NW array. Scale bar: 20 µm. d) SEM image of an (S‐MBA)_2_PbI_4_ NW array with well‐shaped borders and homogeneous width. Scale bar: 10 µm. e) TEM image of a single (S‐MBA)_2_PbI_4_ NW. Scale bar: 1 µm. Inset is the SAED pattern, illustrating the growth of CHP perovskite crystals along the [110] direction. f) AFM image of an individual CHP NW with a height of ≈600 nm and width of ≈3 µm. g) XRD patterns of (R‐, and S‐MBA)_2_PbI_4_ NW arrays showed sharp diffraction peaks, which can be assigned to (002) series of crystal planes. h) The GIWAXS image of (S‐MBA)_2_PbI_4_ NW arrays with highly ordered crystallographic alignment.

**Figure 2:**
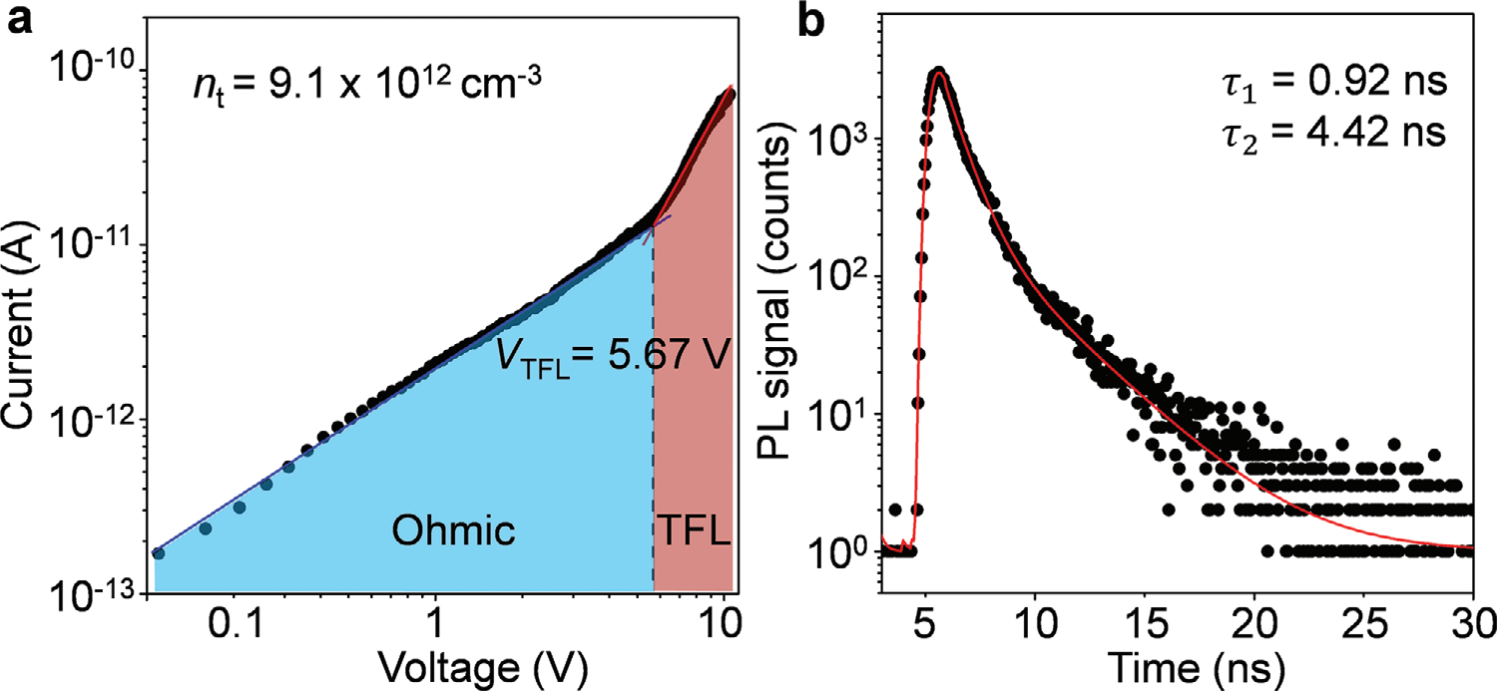
Charge transport efficiency in the CHP single‐crystalline arrays. a) *I*–*V* trace of the (S‐MBA)_2_PbI_4_ NW array, showing two different regions, which are marked for Ohmic (blue region) and TFL (trap‐filled limit, red region). The density of trap state can be extracted from the *V*_TFL_ = 5.67 V. b) PL decay trace of the (S‐MBA)_2_PbI_4_ single‐crystalline NW array. The PL lifetimes are extracted by biexponential fitting with *τ*_1_ = 0.92 ns and *τ*_2_ = 4.42 ns, which are larger than those of polycrystalline thin film (*τ*_1_ = 0.65 ns and *τ*_2_ = 2.23 ns, Figure S11b, Supporting Information), suggesting a longer photocarrier lifetime in CHP single‐crystal NWs.

Here, the (R)‐(+)‐*α*‐methylbenzylamine (R‐MBA) and (S)‐(‐)‐*α*‐methylbenzylamine (S‐MBA) were selected as the chiral organic ligands for the construction of CHPs owing to their chiral amine structure, large *π* bond as well as low cost. The CHP single crystals were first synthesized via a temperature cooling method according to the previous report (Figure S1, Supporting Information).^[^ ^37^ ^]^ **Figure** **1**a presents the crystal structures of (R‐ and S‐MBA)_2_PbI_4_ CHPs, where each inorganic (PbI_6_)^4−^ octahedral layer is sandwiched by two layers of chiral organic chains (R‐MBA or S‐MBA), forming 2‐fold screw symmetry elements and a non‐centrosymmetric chiral space group (*P*2_1_2_1_2_1_),^[^ ^9^ ^]^ which endows the resultant crystals with opposite chirality due to the effective chirality transfer between them.^[^ ^19^, ^37^, ^38^ ^]^ Powder X‐ray diffraction (XRD) analysis was carried out on the ground powders of (R‐, and S‐MBA)_2_PbI_4_ crystals, and all diffraction peaks of the experimental results match well with the simulated results (Figure S2, Supporting Information), indicating the phase purity of the as‐synthesized chiral layered structures. To confirm the chirality of the synthesized CHP, we measured the CD spectra of the (R‐ and S‐MBA)_2_PbI_4_ thin films. Strong CD signals were observed at almost the same wavelengths (at 331, 389, 481, 497, 510 nm) but with oppositely signed values for (R‐ and S‐MBA)_2_PbI_4_ films (Figure 1b). Note that the observed CD positions are significantly different from those of S‐MBA and R‐MBA (255, 261, and 268 nm, Figure S3, Supporting Information), verifying the successful chirality transfer from the organic ligands to the perovskite frameworks. In addition, comparison of the CD spectra and extinction spectra of the chiral perovskite films (**Figure** **2**b,c) revealed that the CD peaks were located before the extinction band edge, whose peak was observed at 497 nm and extrapolated to 523 nm. The anisotropy factor of CD (*g*_CD_) was calculated to be ≈0.0003 at the wavelength of 510 nm according to the formula *g*_CD_ = CD/(32980 × absorbance), where the absorbance was extracted from the extinction spectra (Figure S4, Supporting Information).

The CHP single‐crystalline NW arrays were fabricated from the CHP single‐crystal precursor solution through a micropillar template‐assisted capillary‐bridge rise approach based on an asymmetric‐wettability mechanism (see details in Experimental section and Figure S5, Supporting Information). The utilization of CHP single‐crystal precursor solution is contributed to improving the crystallinity because of the reduced extra impurities or defects caused by the formation of an intermediate phase.^[^ ^39^, ^40^ ^]^ The morphology and crystal structure of (S‐MBA)_2_PbI_4_ single‐crystalline NW arrays were fully investigated (see the Experimental section for details and Figures S6–S8, Supporting Information for the optimization studies). As presented in Figure 1c, the acquired single‐crystalline NW array exhibits uniform and bright green fluorescence under UV light irradiation, indicating its homogeneity and superior crystallinity. The scanning electron microscopy (SEM) image in Figure 1d illustrates a series of perovskite microstructures with a well‐shaped border and homogeneous width assembled in the array. Furthermore, the NWs are of high‐quality crystalline structure growing along the [110] direction, as evidenced by the transmission electron microscopy (TEM) image and the corresponding selected area electron diffraction (SAED) pattern in Figure 1e, which endows them with excellent photoelectric properties. An atomic force microscopy (AFM) image depicts that the as‐fabricated (S‐MBA)_2_PbI_4_ NW with 600 nm height has smooth surfaces (Figure 1f). With template‐induced precise assembly, CHP NW arrays with different sizes and densities can be readily obtained by changing the width and separation of micropillars (Figure S9, Supporting Information).

The crystallographic orientation characterization of NW arrays on a macroscopic scale was performed by XRD analysis. As shown in the XRD patterns of (R‐, and S‐MBA)_2_PbI_4_ single‐crystal NW arrays, sharp diffraction peaks can be assigned to (002), (004), (006), (008), and (0010) lattice planes (Figure 1g), demonstrating the high orientation of the chiral perovskite layers. Benefitting from the utilization of redissolved CHP single crystals precursor solutions, no additional impurity peaks were detected in both systems, (R‐, and S‐MBA)_2_PbI_4_ NWs, which is favorable for realizing chiral semiconductor with high mobility. The degree of crystallographic orientation was further confirmed by the grazing incidence wide‐angle X‐ray scattering (GIWAXS) measurement (Figure 1h). Sharp and discrete Bragg diffraction spots are observed in (S‐MBA)_2_PbI_4_ array, indicating highly ordered crystallographic alignment, which is significantly different from the corresponding thin films (Figure S10, Supporting Information). The improved crystallinity and orientation of CHP NW array could be beneficial to promote the carrier mobility of the photodetector. As shown in Figure S11a, Supporting Information, the absorption spectra of (R‐, and S‐MBA)_2_PbI_4_ NW arrays reveal typical band‐edge absorption at around 530 nm in such chiral perovskites.^[^ ^37^ ^]^ Moreover, the narrow photoluminescence (PL) peaks near the bandgap manifest a low trap density in the perovskite arrays (Figure S11b, Supporting Information).^[^ ^25^, ^26^ ^]^

To evaluate the charge transport efficiency of the acquired CHP NW arrays, we measured the current–voltage (*I*–*V*) trace and analyzed with space charge limited current technique.^[^ ^25^, ^41^, ^42^ ^]^ The *I*–*V* response was ohmic (blue line) at low voltage, while a rapid nonlinear rise named trap‐filled limit (red line) at the *V*_TFL_ = 5.67 V was observed, representing all the available trap states were filled by the injected carriers (Figure 2a). Trap density is an important parameter to examine the electronic properties because defects will capture carriers and thus hinder charge transport. The charge‐trap density of (S‐MBA)_2_PbI_4_ arrays was calculated to be 9.1 × 10^12^ cm^−3^, which is much lower than that of the corresponding thin film (3.4 × 10^16^ cm^−3^, Figure S12a, Supporting Information). This result suggests that the CHP NW single‐crystalline arrays point to a low‐defect electronic structure. Furthermore, the charge carrier lifetime *τ* was measured to estimate the carrier transport properties of the as‐prepared single‐crystal NW arrays with time‐resolved PL (TRPL). The PL decay traces can be well fitted by a bi‐exponential decay model with *τ*_1_ = 0.92 ns and *τ*_2_ = 4.42 ns (Figure 2b), which are larger than those of polycrystalline thin film (*τ*_1_ = 0.65 ns and *τ*_2_ = 2.23 ns, Figure S12b, Supporting Information), suggesting a longer photocarrier lifetime in the CHP NWs than that in corresponding thin‐film. The low charge trap density and long carrier lifetime of CHP NW arrays demonstrate that the charge transport efficiency has been effectively improved in the high‐quality and crystallographic‐ordered microstructures,^[^ ^25^, ^43^ ^]^ which is beneficial for achieving high‐performance CPL detection.

**Figure 3:**
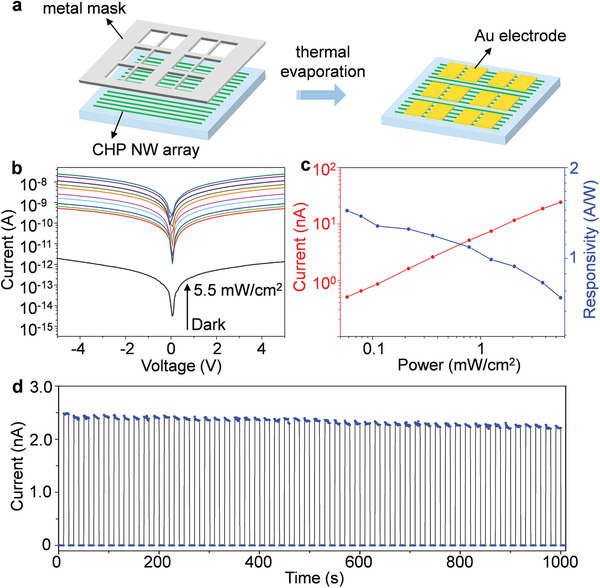
Polarization light response of the CHP NW array‐based photodetectors. a) Schematic illustration of the device fabrication process. Au electrodes are deposited using thermal evaporation with a specific metal mask. b) Dark current and photocurrent under dark and 510 nm LCP illumination with different irradiance of the (S‐MBA)_2_PbI_4_ NW array‐based detector. c) Photocurrent and responsivity of the (S‐MBA)_2_PbI_4_ NW array device under different incident powers. d) *I*–*t* response of the (S‐MBA)_2_PbI_4_ NW array photodetector at 510 nm illumination and 5 V bias, verifying the excellent photo‐switching behavior of the device.

The optoelectronic performances of the synthesized (R‐, and S‐MBA)_2_PbI_4_ NW arrays were studied by fabricating CPL photodetectors with a lateral configuration. Au electrodes defining a channel length of 20 µm were deposited using thermal evaporation with a specific metal mask (**Figure** **3**a). The LCP and RCP as the illumination source are generated by a linear polarizer coupled with a quarter‐wave plate (Figure S13, Supporting Information). The 510 nm laser was chosen as the excitation light because the CHP has good absorbing ability and strong CD intensity at this wavelength. Figure 3b shows a representative set of the *I*–*V* curves of (S‐MBA)_2_PbI_4_‐based photodetector under dark and 510 nm LCP illumination with varied incident power. This array‐based device presents a typical low dark current of 1.36 × 10^−12^ A at 5 V bias, which was associated with its intrinsic low free carrier density characteristics. Light on/off ratio of 1.8 × 10^4^ was calculated by extracting the current value under dark and 510 nm LCP illumination at 5.5 mW cm^−2^, which is about three order of magnitude higher than that of generally obtained film device (36.8, Figure S14a, Supporting Information), indicating low noise current and high light responsivity of CHP NW array device.

The incident power‐dependent photocurrent and responsivity of (S‐MBA)_2_PbI_4_ array photodetector were plotted in Figure 3c. The photocurrent (*I*_ph_) of devices can be calculated by *I*_ph_ = *I*_light_ − *I*_dark_, where *I*_light_ and *I*_dark_ are the currents under light illumination and dark, respectively. The responsivity (*R*) is given by *R* = *I*_ph_/*P*, where *P* is the illuminant power, reflecting the response sensitivity of the device to incident light. Under irradiance of 0.06 mW cm^−2^, the highest responsivity of this device can reach 1.44 A W^−1^. This value is nearly one order of magnitude larger than that of the CHP film‐based CPL photodetectors (Figure S14b, Supporting Information). Such high photocurrent and responsivity can be ascribed to the long carrier lifetime and low charge trap density in single‐crystal structures. The highest D* reached up to 3.9 × 10^12^ Jones at a low light power density of 0.06 mW cm^−2^ (Figure S15a, Supporting Information), which is higher than the reported value of chiral perovskite film photodetectors.^[^ ^19^ ^]^ The responsivities of (S‐MBA)_2_PbI_4_ array‐based photodetector also exhibit obvious voltage dependence at various light power densities (Figure S15b, Supporting Information). The −3 dB frequency, which is defined as the frequency where the response dropped to half of the initial value, was about 500 Hz for 510 nm response, indicating the fast response speed of single‐crystal nanowire devices (Figure S16, Supporting Information). As shown in Figure 3d, the *I*–*t* temporal response of the (S‐MBA)_2_PbI_4_ NW array photodetector is evaluated at 510 nm illumination and 5 V bias with a time interval of 10 s for 1000 s (50 ON/OFF cycles), verifying the excellent photo‐switching behavior of the device. Moreover, (R‐MBA)_2_PbI_4_ NW array‐based photodetector also exhibits high photocurrent and light on/off ratio (Figure S17, Supporting Information), which is in consistent with that of (S‐MBA)_2_PbI_4_ array device and validates the superior optoelectronic performance of the CHP single‐crystalline arrays. In addition, the CHP NW array photodetectors with various heights and widths exhibited excellent photoresponse performance, indicating no obvious size dependence (Figure S18, Supporting Information).

**Figure 4:**
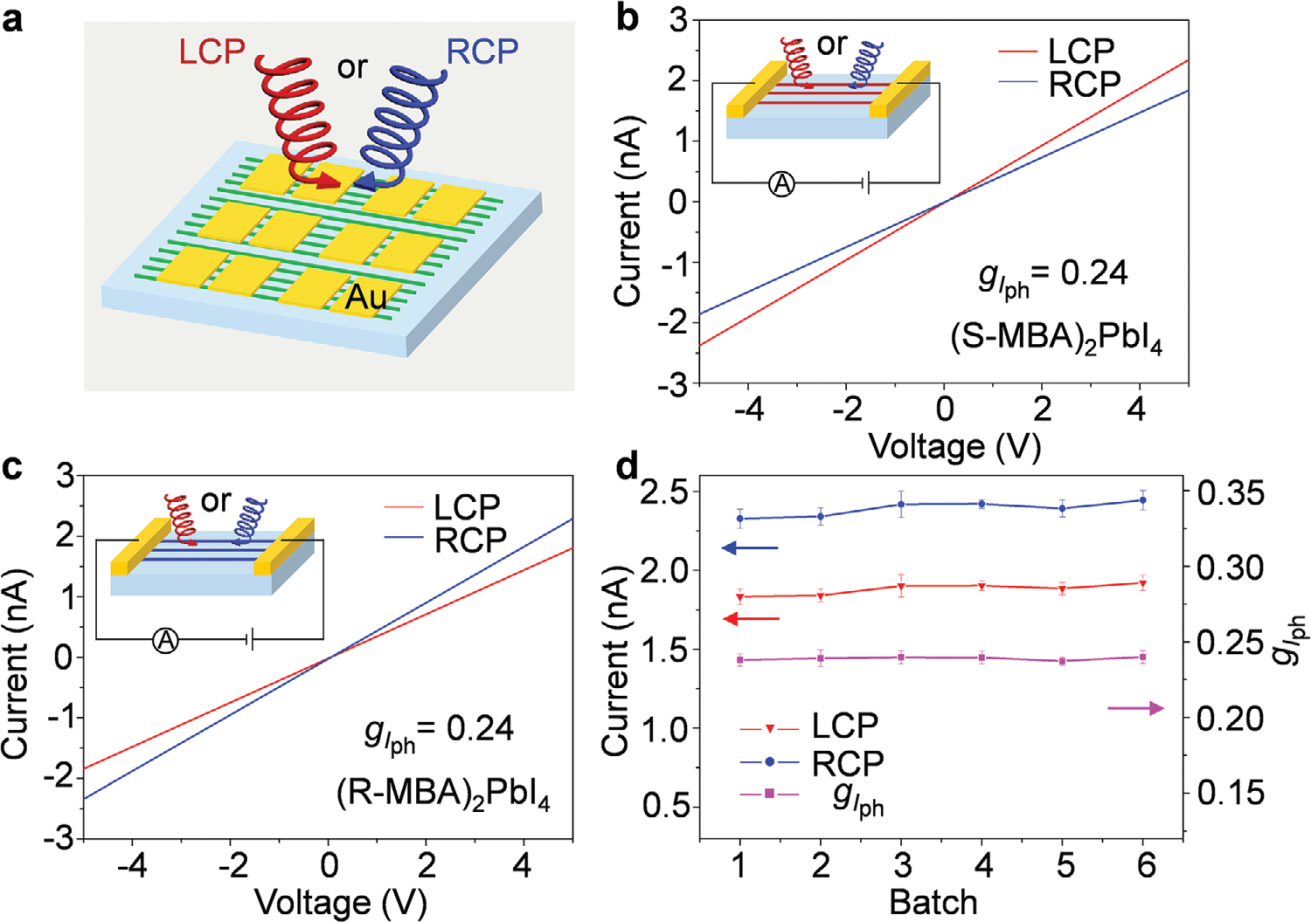
Chiral discrimination of the CHP NW array‐based photodetectors. a) Schematic illustration of the CPL photodetector based on CHP single‐crystalline NW arrays. b,c) *I*–*V* characteristics and the calculated *g_I_*_ph_ of (S‐MBA)_2_PbI_4_ and (R‐MBA)_2_PbI_4_ array‐based detectors under CPL illumination (*λ* = 510 nm, power = 0.32 mW cm^−2^), evidencing significant CPL distinguishability. d) Statistical photocurrent and *g_I_*_ph_ of 30 (R‐MBA)_2_PbI_4_ array‐based detectors from different batches. Error bars represent the standard deviation of five representative measurements from the same batch under CPL illumination (*λ* = 510 nm, power = 0.32 mW cm^−2^).

The chiral discrimination performance of the photodetectors was further investigated by measuring their photocurrents under different CPL irradiation (**Figure** **4**a). Before measurement, we carefully calibrated the whole system to make sure that the intensity of LCP and RCP illumination was the same during all measurements (Figure S19, Supporting Information). The *I*–*V* characteristics of (R‐, and S‐MBA)_2_PbI_4_ photodetectors under 510 nm CPL irradiations at an optical power density of 0.32 mW cm^−2^ are shown in Figure 4b,c. The (S‐MBA)_2_PbI_4_ array device exhibits a larger photocurrent under the LCP illumination compared with the RCP illumination, demonstrating significant CPL distinguishability (Figure 4b). In order to quantify the distinguishability of CPL detection, we define the anisotropy factor for photocurrent (*g_I_*_ph_) as:

(1) $$g_{I_{\mathrm{ph}}}=2\frac{I_{\mathrm{ph}}^{R}-I_{\mathrm{ph}}^{L}}{I_{\mathrm{ph}}^{R}+I_{\mathrm{ph}}^{L}}$$

where *I* ^L^_ph_ and *I* ^R^_ph_ represent the photocurrent under LCP and RCP illumination. The *g_I_*_ph_ of (S‐MBA)_2_PbI_4_ NW array‐based detector is calculated to be 0.24 at 5 V bias voltage, which is larger than that of the corresponding thin‐film photodetector (*g_I_*_ph_ = 0.07, Figure S20, Supporting Information). The increased *g_I_*_ph_ of CHP NW array‐based detector might be attributed to the long spin lifetimes originated from their excellent crystalline nature.^[^ ^44^, ^45^ ^]^ Although the *g*_CD_ at the wavelength of 510 nm is relatively low, the single‐crystal array achieved a high *g_I_*_ph_ due to its high crystallinity and ordered arrangement, which greatly improves the charge transport efficiency compared with polycrystalline thin‐film.^[^ ^29^ ^]^

Similar chiral discrimination capability was observed in the (R‐MBA)_2_PbI_4_ arrays‐based detector, where the photocurrent under LCP irradiation is smaller than that obtained from RCP irradiation with *g_I_*_ph_ = 0.24 (Figure 4c). In contrast, racemic CHP ((rac‐MBA)_2_PbI_4_) NW array‐based photodetector does not show any photocurrent differences between RCP and LCP illumination (Figure S21, Supporting Information), manifesting that the polarization distinguishability is originated from the introduction of homochiral organic ligands. Moreover, we further explore the reliability and reproducibility of NWs device by measuring the CPL detecting performance of five devices from six different batches. As shown in Figure 4d, multiple devices composed of (R‐MBA)_2_PbI_4_ NWs exhibit similar photocurrents under CPL illumination, and the calculated values of *g_I_*_ph_ also show negligible distinction, revealing excellent reliability of CHP single‐crystal arrays for high‐performance CPL detection. Additionally, the XRD pattern and photoresponse of (S‐MBA)_2_PbI_4_ array were further monitored after being stored in ambient without any encapsulation for 1 week. These characteristics show no significant difference after the ambient storage, demonstrating the long‐term performance of these array‐based devices (Figure S22, Supporting Information).

## 3 Conclusion

In summary, we have successfully demonstrated a high‐performance CPL photodetector with (R‐, and S‐MBA)_2_PbI_4_ single‐crystalline arrays. Through a micropillar template‐assisted capillary‐bridge rise approach, large‐scale CHP single‐crystal NWs with high crystallinity and ordered crystallographic alignment were fabricated. The reduced charge trap density and extended carrier lifetimes in these CHP microstructures enable the promoted charge transport efficiency. The corresponding CHP array‐based CPL photodetector shows excellent optoelectronic performance with high light on/off ratio of 1.8 × 10^4^, and the responsivity up to 1.4 A W^−1^. This device exhibits high distinguishability with an outstanding anisotropy factor of 0.24 for photocurrent, which can be attributed to the long spin lifetimes originated from their excellent crystalline nature. We believe these results are likely to provide a new avenue to effectively promote the practical application of chiral optoelectronic devices.

## Conflict of Interest

The authors declare no conflict of interest.

## Supporting information

Supporting Information

## Data Availability

The data that support the findings of this study are available on request from the corresponding author.
