# Supplementary material for: Chiral Hybrid Perovskite Single‐Crystal Nanowire Arrays for High‐Performance Circularly Polarized Light Detection
Source: Adv Sci (Weinh). 2021 Sep 24;8(21):2102065. doi: 10.1002/advs.202102065 (PMC8564458; doi:10.1002/advs.202102065)
Supplement: Supplementary file 1 — Supporting Information [file ADVS-8-2102065-s001.pdf]

© 2021 Wiley-VCH GmbH

ADVANCED SCIENCE
Open Access

Supporting Information

for *Adv. Sci.,* DOI: 10.1002/advs.202102065

Chiral hybrid perovskite single-crystal nanowire arrays for high-performance circularly polarized light detection

Zhen Liu, Chunhuan Zhang, Xiaolong Liu, Ang Ren, Zhonghao Zhou, Chan Qiao, Yuwei Guan, Yuqing Fan, Fengqin Hu, and Yong Sheng Zhao**

Supporting Information

Chiral hybrid perovskite single-crystal nanowire arrays for high-performance circularly polarized light detection

Zhen Liu, Chunhuan Zhang, Xiaolong Liu, Ang Ren, Zhonghao Zhou, Chan Qiao, Yuwei Guan, Yuqing Fan, Fengqin Hu, and Yong Sheng Zhao**

College of Chemistry, Beijing Normal University, Beijing 100875, China

Key Laboratory of Photochemistry, Institute of Chemistry, Chinese Academy of Sciences, Beijing 100190, China

University of Chinese Academy of Sciences, Beijing 100049, China

E-mail: fqhu@bnu.edu.cn; yszhao@iccas.ac.cn

Experimental Section

Materials

(R)-(+)-α-methylbenzylamine (R-MBA, 99%, optical purity > 97%), (S)-(-)-α-methylbenzylamine (S-MBA, 98%, optical purity > 97%), Lead (II) acetate trihydrate (99.5%) and 55-57% aqueous hydriodic acid (HI) solution were purchased from Innochem Science & Technology (Beijing, China). N, N-anhydrous dimethylformamide (DMF), anhydrous dimethyl sulfoxide (DMSO), were purchased from J&K Chemical.

Preparation

Synthesis of chiral hybrid perovskite (CHP) single crystals. The synthesis of the CHP single crystals was adapted from literature reports.[1] First, 0.759 g of $(CH_3COO)_2Pb$ (2 mmol), 0.485 g (4 mmol) of R-MBA (for $(R\text{-}MBA)_2PbI_4$ CHP, S-MBA for $(S\text{-}MBA)_2PbI_4$ CHP), and 10 ml of 57% HI solution were loaded into a glass vial. The as-formed yellow precipitates were subsequently dissolved at 100 °C in an oil bath. The reaction stock solution was then cooled to room temperature under ambient conditions, resulting in the formation of orange needle-like crystals. These crystals were vacuum-filtrated and rinsed with toluene several times. Finally, the product was dried in vacuum overnight.

Preparation of CHP polycrystalline films. Quartz or glass substrates were washed sequentially by acetone and isopropanol in an ultrasonic cleaner for 30 min, respectively, followed by oxygen plasma treatment for 10 min. The as-synthesized CHP crystals were dissolved in DMF with a certain concentration (20 wt%) as the precursor solution, and the continuous thin films were prepared on substrates by a spin-coating method at 2000 rpm for 30 s. Finally, the spin-coated film was annealed at 100 °C for 10 min on a hot-plate to induce crystallization.

Selective modification of micropillar template with asymmetric wettability. The templates with periodic micropillar structures of 15 μm in height were fabricated by a direct laser-writing apparatus (Heidelberg DWL200) and a deep reactive-ion etching system (DRIE, Alcatel 601E). The width and separation of micropillars can be controlled by setting up different parameters for photolithography processing. Before modification, the micropillar templates were cleaned with deionized water, ethanol, and isopropanol followed by oxygen plasma for the removal of the contaminants on the surface. To selectively modify the topographical templates into asymmetric wettability, a cured PDMS layer was pressed onto the tops of micropillars. Then the micropillar template covered by PDMS was put into a sealed glass culture dish, which was added 20 μL heptadecafluorodecyltrimethoxysilane (FAS) liquid. After being heated at 90 °C for 6 h, the exposed sidewalls of micropillars were silanized by FAS molecules, resulting in a micropillar template with lyophilic tops and lyophobic sidewalls.

Fabrication of CHP single-crystalline nanowire (NW) arrays. The CHP precursor solution was prepared by directly dissolving synthesized CHP crystals into a mixed solvent of DMF and DMSO (the volume ratio is 1:1). A "capillary-bridge rise" assembly system was constructed by combining an asymmetric-wettability topographical template and a target substrate.[2] The assembly system was contacted with the solution of CHP precursor for a few seconds (Note that the duration will affect the amount of inhaled solution, which will ultimately affect the thickness of the sample). The CHP precursor liquid will rise in the gaps between the top of micropillars and substrate driven by capillary force and Laplace pressure, and then forming individual capillary bridges anchored onto the top of the micropillars. The assembly system was moved away from precursor liquid and heated at 75 °C for 6 hours to

realize the total evaporation of DMF and DMSO solvents. At last, the CHP single-crystalline NWs were generated onto target substrates after detaching the micropillar template.

Characterization

The transmission circular dichroism (CD) data were collected using a CD spectrometer (J-810, JASCO). The morphology and crystallinity of the as-prepared CHP single-crystalline NW arrays were examined by scanning electron microscopy (SEM, Hitachi S-4800), transmission electron microscopy (TEM, JEOL 2100F), and atomic force microscopy (AFM, Bruker MultiMode 8), respectively. Fluorescence micrographs were taken with an inverted fluorescence microscope (Nikon Ti-U) equipped with a high-resolution color digital camera (DS-Ri1). The crystal structure of the as-prepared CHP powders and arrays were characterized by X-ray diffraction (XRD, Japan Rigaku D/max-2500) with Cu Kα radiation (λ = 0.154 nm). The grazing incidence wide-angle X-ray scattering (GIWAXS) was performed on XEUSS SAXS/WAXS system at the incidence angle of 0.2°. The UV-vis absorption and photoluminescence (PL) spectra were measured with a Shimidazu UV-2600 spectrophotometer and spectrometer equipped with a 600 gr/mm granting excited by a 405 nm continuous-wave (CW) laser, respectively. PL lifetime measurements of CHP NW arrays and spin-coated thin film were performed on a Quantaurus-Tau compact fluorescence lifetime spectrometer (Hamamatsu Photonics, C11367-31, Quantaurus-Tau, Japan).

Device fabrication and detector performance

Single-crystalline NW arrays and thin-film devices. For photodetector fabrication, the glass substrate was cleaned with water, ethanol, and acetone. The substrates were subjected to oxygen plasma treatment for 10 min before use. CHP single-crystalline NW arrays were fabricated onto the glass substrate using the asymmetric-wettability assembly method. And

CHP thin film was fabricated onto the glass substrate by spin-coating method. Then, 100 nm Au was deposited by thermal evaporation with a specific metal mask. The channel length and width were controlled to be 20 μm and 200 μm, respectively.

Detector performance measurements. The Current-Voltage (*I-V*) measurements were carried out using a Keithley 4200 semiconductor characterization system and a two-probe system at room temperature. To measure the CPL response of the photoconductor, 510 nm linearly polarized laser was converted to circularly polarized laser light by using a polarizer and quarter-wave plate. The power of the laser was measured with a power meter.

Trap density measurements. We carried out the space charge limited current (SCLC) analysis to evaluate the charge-carrier trap density in CHP single-crystalline NW arrays and spin-coated thin-films. A vertical-structure device for thin-film was employed by sandwiching the CHP layer between ITO layer and Au layer for applying an electric field. The fabrication of ITO/(S-MBA)$_2$PbI$_4$ thin film /Au structure devices is to first spin-coat CHP solution on an ITO substrate and then evaporate 20 nm Au with a specific metal mask. The NW arrays devices were fabricated in lateral configuration with a channel length of 20 μm. Representative logarithmic *I-V* traces of CHP single-crystalline NWs and polycrystalline films are shown in Figure 3a and Figure S11a, demonstrating a clear transition from Ohmic regime ($I \sim V_m$, m ≈ 1) to trap filling limited (TFL) regime ($I \sim V_m$, m > 3). The trap density was calculated according to the following equation:

$$n_t = \frac{2\varepsilon\varepsilon_0 V_{TFL}}{ed^2}$$

where ε and ε_0 are the relative and vacuum dielectric constants, respectively, *e* the elementary charge and d the length of conductive charge (*d* is the thickness of the material for thin-film device, and channel length for NW array device).

Statistical Analysis

Multiple devices were tested by Keithley 4200 to extract current under CPL illumination (λ = 510 nm, power = 0.32 mW/cm^2, Figure 4d). The sample size (n) for each statistical analysis was chosen as 5. Origin software was used to calculate the standard deviation and mean value for statistical analysis. Error bars represent the standard deviation of five representative measurements from the same batch.

Multiple light intensities were monitored by a power meter (Figure S19). The sample size (n) for each statistical analysis was chosen as 50. Origin software was used to calculate the standard deviation and mean value for statistical analysis.

Supplementary Figures

Figure S1. Photographs of CHP single crystals taken at different stages of the synthesis process.

Figure S2. Experimental and simulated powder XRD patterns of (R- and S-MBA)$_2$PbI$_4$.

Figure S3. The CD spectra of R-MBA and S-MBA chiral ligands.

Figure S4. Normalized extinction spectra of (S-MBA)$_2$PbI$_4$ and (R-MBA)$_2$PbI$_4$ films.

Figure S5. Schematic illustration of the preparation of CHP single-crystalline NW arrays.

Figure S6. Fluorescence microscopy images of the CHP single-crystalline NW arrays fabricated from different solvents.

Figure S7. Fluorescence microscopy images of the CHP single-crystalline NW arrays fabricated with different precursor concentrations.

Figure S8. Fluorescence microscopy images of the CHP single-crystalline NW arrays fabricated with different temperatures.

Figure S9. Designable fabrication of the CHP single-crystalline NW arrays with different line widths and densities.

Figure S10. Crystallographic orientation and morphology characteristics of (S-MBA)$_2$PbI$_4$ films.

Figure S11. UV–vis absorption and photoluminescence spectra of (R- and S-MBA)$_2$PbI$_4$ single-crystalline NW arrays.

Figure S12 Charge transport efficiency in CHP thin-film.

Figure S13. Schematic illustration of the home-built two-probe micro-photoelectric testing system.

Figure S14. Polarization light response of the CHP film-based photodetector.

Figure S15. Detectivity and voltage-dependent performances of (S-MBA)$_2$PbI$_4$ NW array.

Figure S16. Normalized responses of chiral perovskite nanowire devices at various frequencies.

Figure S17. Current response of the (R-MBA)$_2$PbI$_4$ NW array under dark and RCP illumination.

Figure S18. Responsivities of CHP NW array with different heights and widths.

Figure S19. The intensity calibration of LCP and RCP illumination.

Figure S20. *I-V* curves of (S-MBA)$_2$PbI$_4$ film under 510 nm RCP and LCP illumination.

Figure S21. *I-V* curves of the (rac-MBA)$_2$PbI$_4$ NW array under 510 nm RCP and LCP illumination.

Figure S22. The ambient stability of (S-MBA)$_2$PbI$_4$ array.

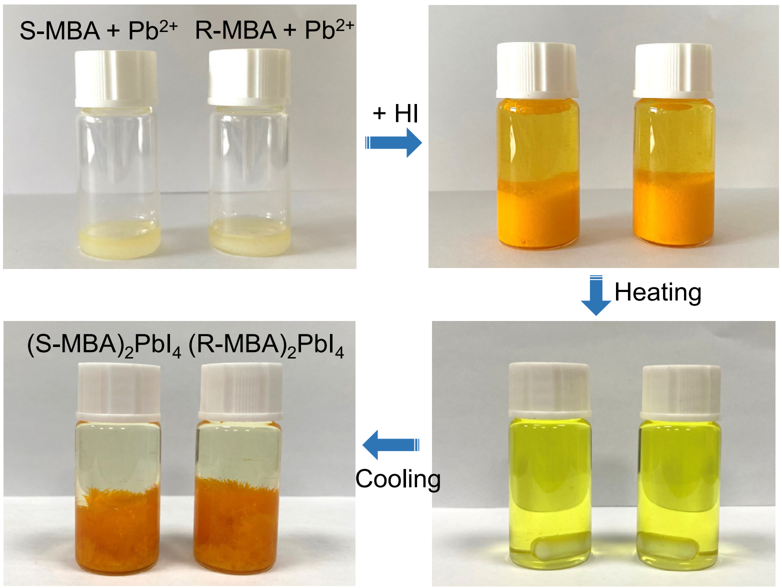

Figure S1. Photographs of CHP single crystals taken at different stages of the synthesis process.

As shown in Figure S1, the photographs indicate that the end products via a temperature cooling method are orange needle-like crystals, which are obviously distinguish from as-formed yellow precipitates. The obviously change of the product morphology indicates that the chiral hybrid perovskites have been successfully synthesized. The CHP precursor solution prepared by synthesized CHP crystals could be beneficial to obtain high-quality single-crystlline array.

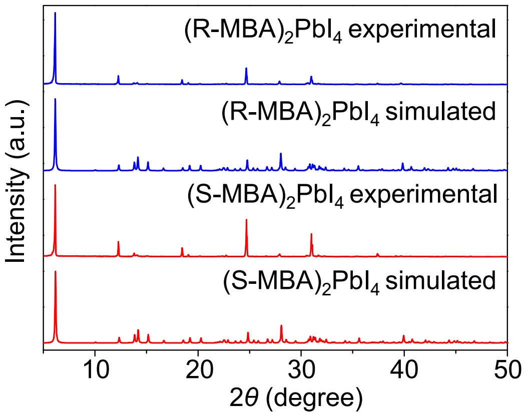

Figure S2. Experimental and simulated powder XRD patterns of (R- and S-MBA)$_2$PbI$_4$.

As shown in Figure S2, the diffraction peaks in the powder X-ray diffraction (XRD) pattern of the CHPs are narrow and strong, implying their high crystallinity. Meanwhile, all the diffraction peaks of (R- and S-MBA)$_2$PbI$_4$ powder well match those of the simulated results from the single crystal diffraction, indicating the high phase purity of the as-synthesized chiral layered structures. Such CHPs with excellent crystallinity and purity would be conducive to acquire high-quality CHP single-crystal arrays.

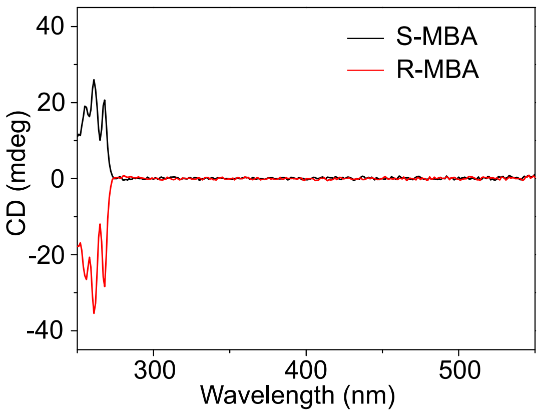

Figure S3. The CD spectra of R-MBA and S-MBA chiral ligands.

R-MBA and S-MBA were diluted in ethanol solution with a volume ratio of 1:1000. The CD peaks of R-MBA and S-MBA were at 255 nm, 261 nm and 268nm, which are different from the CD peaks of (S- and R-MBA)$_2$PbI$_4$ films, verifying the successful chirality transfer from the organic ligands to the perovskite frameworks.

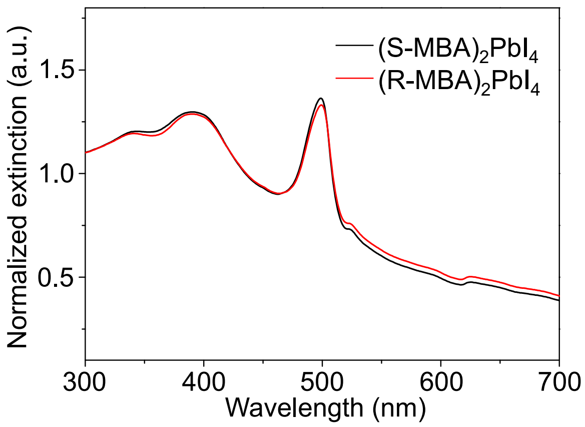

Figure S4. Normalized extinction spectra of $(S\text{-}MBA)_2PbI_4$ and $(R\text{-}MBA)_2PbI_4$ films.

Comparing to the CD spectra in Figure 1b, extinction spectra of the chiral perovskite films revealed that the CD peaks were located before the extinction band edge (524 nm), which is consistent with the result in previous paper.[1] The value of the absorption was used to calculate the anisotropy factor of CD (g_{CD}).

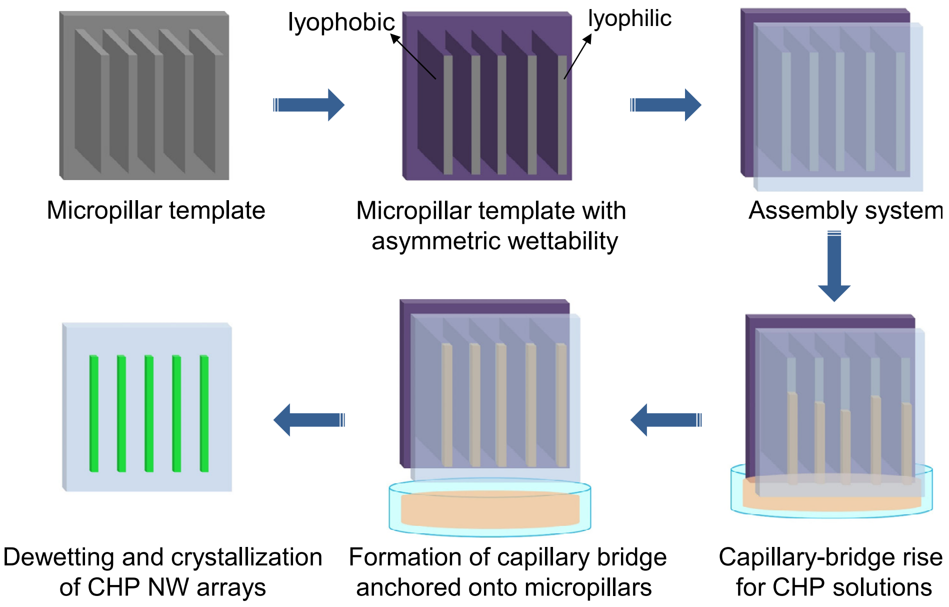

Figure S5. Schematic illustration of the preparation of CHP single-crystalline NW arrays.

As shown in Figure S5, the CHP single-crystalline NW arrays were fabricated through a universal and facile micropillar template-assisted capillary-bridge rise approach with an asymmetric-wettability mechanism.[2] The as-synthesized (R-, and S-MBA)$_2$PbI$_4$ crystals were firstly dissolved in solvent to serve as precursor solutions for subsequent preparation process. To generate ordered single-crystalline chiral perovskite arrays, an assembly system was constructed by combining an asymmetric-wettability topographical template and a target substrate. The assembly system was contacted with the CHP precursor solution for a few seconds. Afterward, the CHP precursor solution would rise in the gaps between the top of micropillars and substrate driven by capillary force and Laplace pressure, thus forming individual capillary bridges anchored onto the top of the micropillars. CHP single-crystalline NW arrays were finally generated on the predetermined positions through a dewetting process.

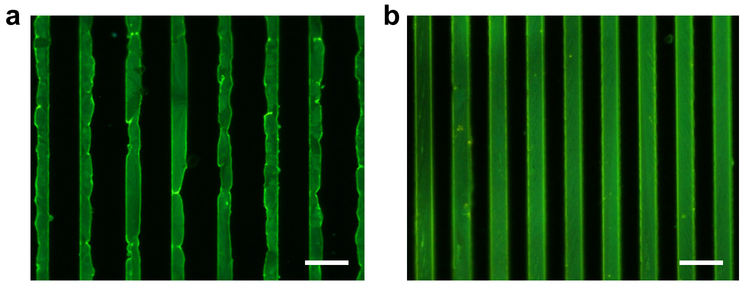

Figure S6. Fluorescence microscopy images of the CHP single-crystalline NW arrays fabricated from different solvents. a) DMF; b) DMF/DMSO mixed solvent (the volume ratio is 1:1). Scale bars: 10 μm.

The solvent used in the fabrication process of CHP single-crystalline NW arrays is a crucial factor that will influence the crystal growth of NWs. As shown in Figure S6a, as-prepared CHP NW arrays with pure DMF solvent show rough surfaces and discontinuous crystallization due to the fast crystallization rate during the drying process. The addition of DMSO solvent could improve the crystallization quality by lowering the crystallization rate because DMSO has a higher coordination affinity, a relatively high boiling point (189 °C) and a low saturated vapor pressure (Figure S6b). It demonstrates that the DMF/DMSO mixed solvent is beneficial to the preparation of high-quality CHP NW arrays toward high-performance CPL detection.

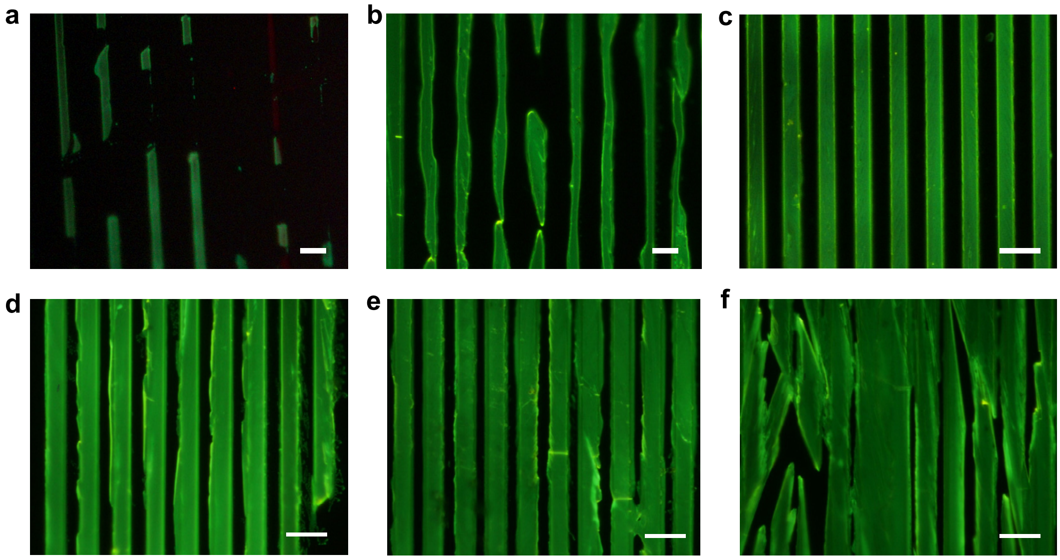

Figure S7. Fluorescence microscopy images of the CHP single-crystalline NW arrays fabricated with different precursor concentrations varying from a) 0.01 mol L^{-1}, b) 0.05 mol L^{-1}, c) 0.1 mol L^{-1}, d) 0.5 mol L^{-1} and e) 1 mol L^{-1} to f) 2 mol L^{-1}. Scale bars: 10 μm.

The precursor concentration is essential to the crystal growth of CHP single-crystalline NW arrays. As displayed in Figure S7, discontinuous microbelts are generated at excessively low concentrations, while the incomplete film is fabricated at high concentrations. The optimal precursor concentration was determined to be 0.1 mol/L for the synthesis of high-quality CHP single-crystalline NW array.

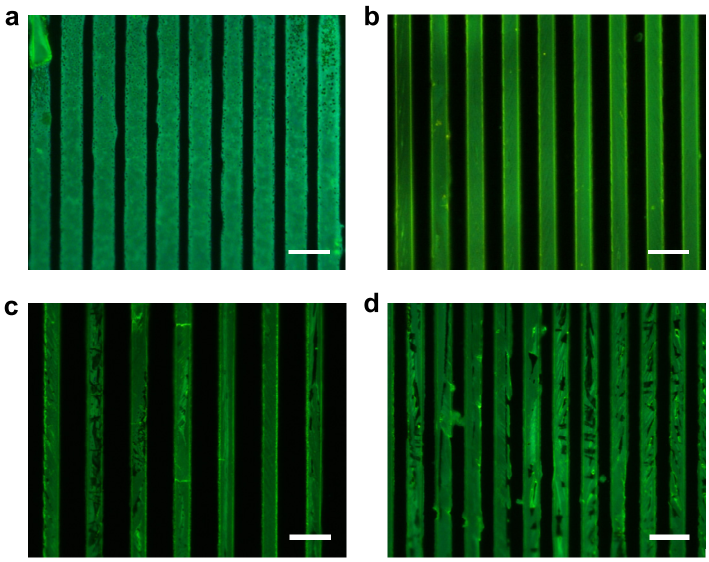

Figure S8. Fluorescence microscopy images of the CHP single-crystalline NW arrays fabricated with different temperatures of a) 60 °C, b) 75 °C, c) 90 °C, and d) 120 °C. Scale bars: 10 μm.

The crystallization behavior of chiral perovskite arrays is also related to the temperature, with the other conditions being the same. As shown in Figure S8a, the green liquid film indicates that the precursor concentration has not completely evaporated within 6 hours when the temperature is 60 °C. And when the temperature is as high as 90 °C or above, CHP arrays show incomplete crystal growth due to the rapid crystallization process (Figure S8c-d). These results imply that an appropriate temperature (75 °C) is favorable for the preparation of CHP NW arrays with high crystallinity.

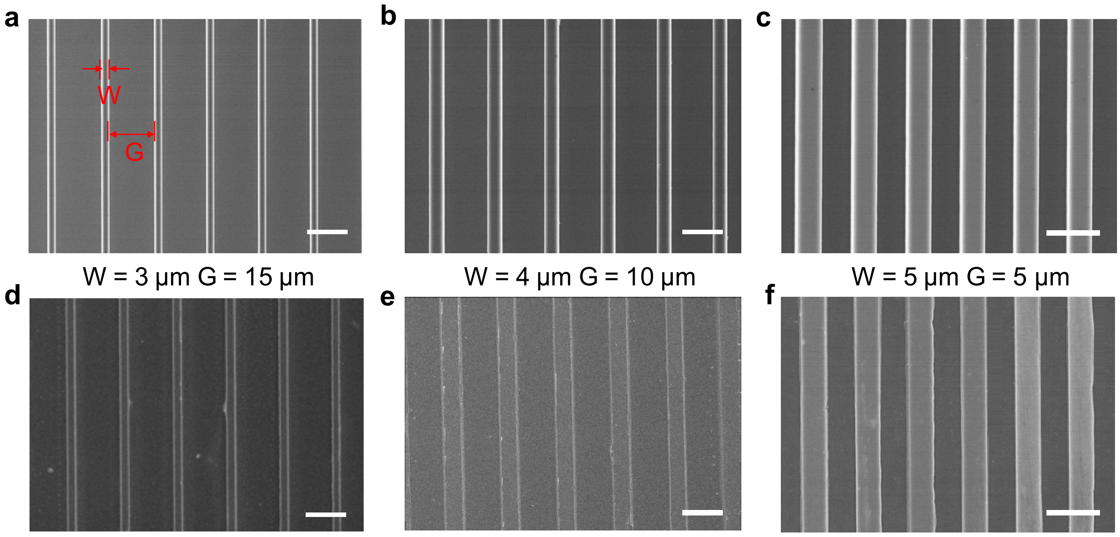

Figure S9. Designable fabrication of the CHP single-crystalline NW arrays with different line widths and densities by changing the parameters of micropillar templates. a-c) SEM images of templates with different parameters. d-f) SEM images of the NW arrays fabricated with the corresponding templates in a-c, respectively. Templates' parameters: a), 3 μm width (W) and 15 μm gap (G), b), 4 μm width and 10 μm gap, c), 5 μm width and 5 μm gap. Scale bars: 10 μm.

As shown in the Figure S9, the NW arrays with different line widths and densities can be readily obtained by changing the width and spacing of micropillars, proving that the micropillar templates can guide the precise assembly of CHP NW arrays with desirable pattern.

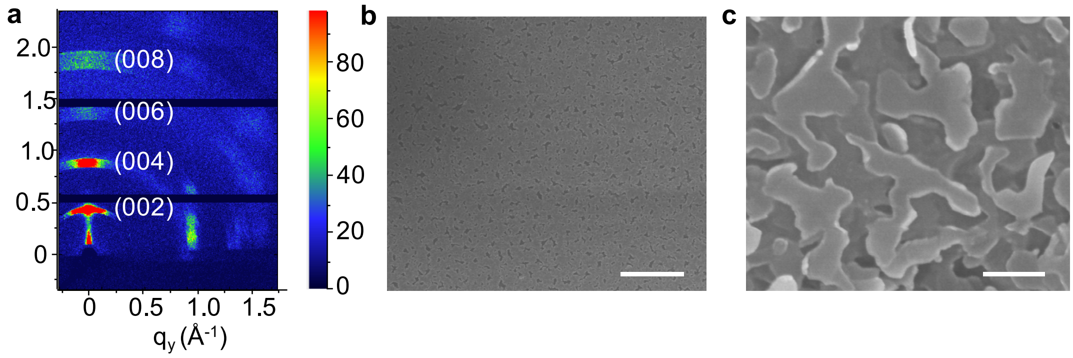

Figure S10. Crystallographic orientation and morphology characteristics of $(S\text{-}MBA)_2PbI_4$ films. a) GIWAXS pattern, b) SEM image and c) Zoom-in SEM image of $(S\text{-}MBA)_2PbI_4$ film. Scale bars: b) 100 μm and c) 400 nm.

As shown in the Figure S10a, dispersive diffraction rings that could be assigned to the diffraction from (002*l*) peaks were observed from the $(S\text{-}MBA)_2PbI_4$ film, indicating the film has more random orientation and weaker crystallinity than CHP single-crystalline NW arrays. SEM image and Zoom-in SEM image demonstrate thin film is polycrystalline with ubiquitous grain boundaries and hundred-nanometer crystallites, which will lead to low photodetection performances. In contrast, the crystallinity and orientation of CHP single-crystal arrays have been obviously improved compared to these polycrystalline films, which is favorable for constructing high-performance CPL detectors.

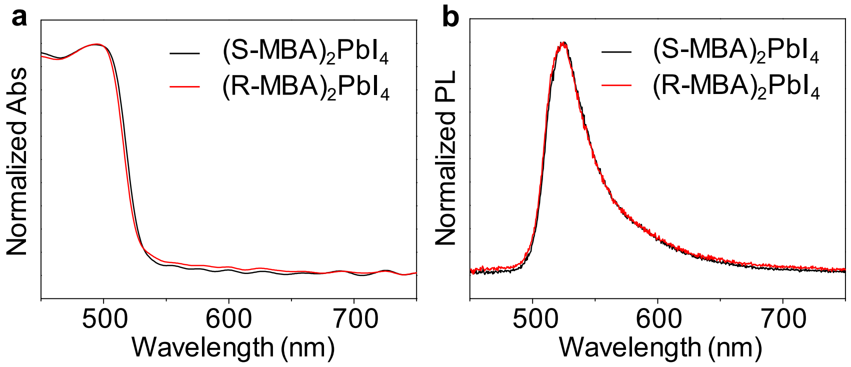

Figure S11. a) UV–vis absorption spectra b) photoluminescence spectra of (R- and S-MBA)$_2$PbI$_4$ single-crystalline NW arrays.

Figure S11a displays the room-temperature UV−vis absorption spectra of the as-synthesized (R- and S-MBA)$_2$PbI$_4$ single-crystalline NW arrays, revealing typical band-edge absorption behavior at around 530 nm in such chiral perovskite.[1] Photoluminescence (PL) spectrum of the CHP NW arrays excited by a linearly polarized 405 nm CW laser is showed in Figure S11b. A low trap density in the NW arrays was manifested by the narrow PL peak near the bandgap, which benefits the preparation of high-performance CPL optoelectronic devices.

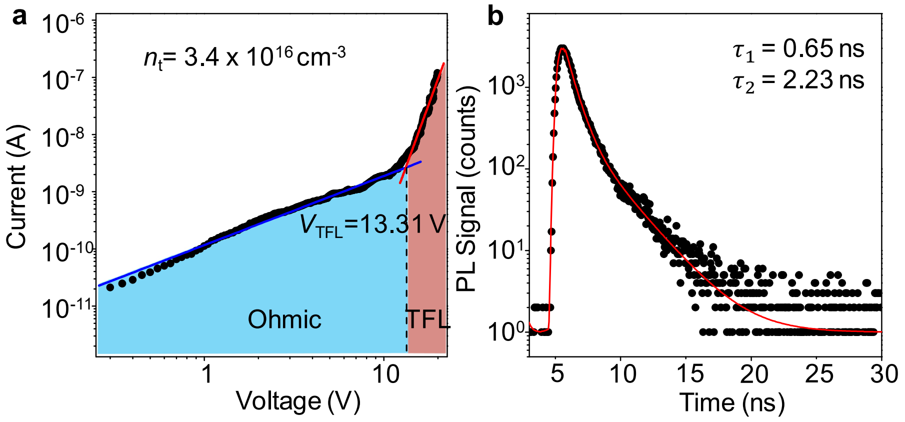

Figure S12. Charge transport efficiency in CHP thin-film. a) *I-V* trace and b) PL lifetime of the $(S\text{-}MBA)_2PbI_4$ film.

The *I-V* trace and PL lifetime were measured to compare the charge transport efficiency between CHP single-crystalline NW array and the corresponding thin-film. According to the previously mentioned equation, the trap density of the $(S\text{-}MBA)_2PbI_4$ film was calculated to be 3.4×10^{16} cm^{-3}, noticeably higher than that of $(S\text{-}MBA)_2PbI_4$ NW array in Figure 2a. The PL lifetimes were extracted by bi-exponential fitting with $\tau_1 = 0.65$ ns and $\tau_2 = 2.23$ ns, which is smaller than that of NW array ($\tau_1 = 0.92$ ns and $\tau_2 = 4.42$ ns) in Figure 2b, suggesting that the CHP NWs are expected to possess a longer photocarrier lifetime than the corresponding thin-film. Therefore, the charge transport efficiency has been effectively improved in high-crystallinity and crystallographic-ordered CHP array,[3] making them more promising for achieving high-performance CPL detection.

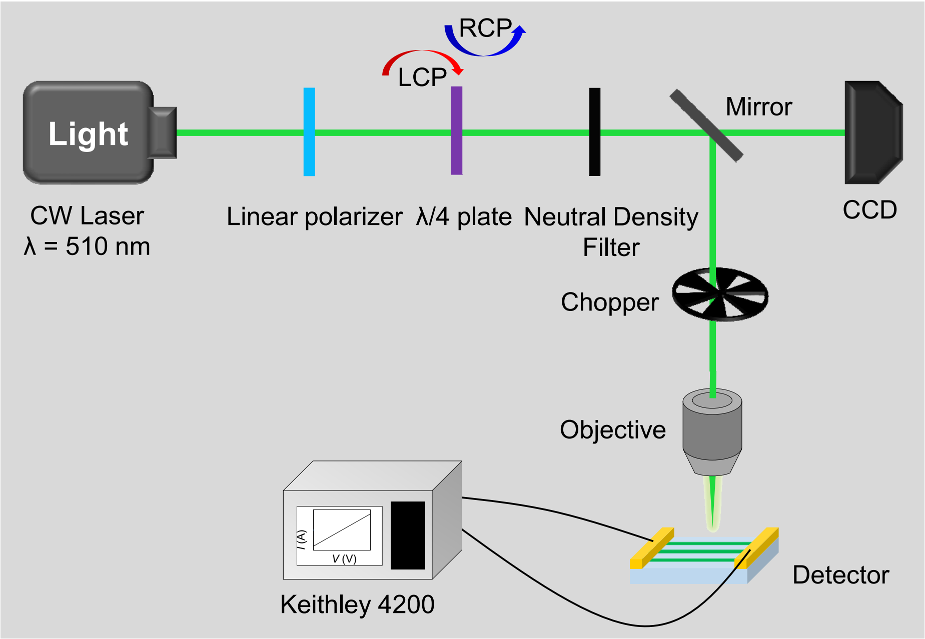

Figure S13. Schematic illustration of the home-built two-probe micro-photoelectric testing system.

Circularly polarized light detection was investigated with a home-built two-probe micro-photoelectric testing system. The light source is a CW laser (λ = 510 nm) with tunable illumination intensity. The left-handed polarized light (LCP) and right-handed polarized light (RCP) are generated by a linear polarizer coupled with a quarter-wave plate. The sample on a glass substrate (refractive index ~1.5) was excited with CPL laser beams through an objective (Nikon CFLU Plan, 20, N.A. = 0.4), with input power altered by neutral density filters. The chopper was used to modulate the CW laser into a periodically discontinuous laser with a certain frequency. The characteristics of photodetectors were investigated by using a Keithley 4200 SCS semiconductor parameter analyzer with a two-probe station.

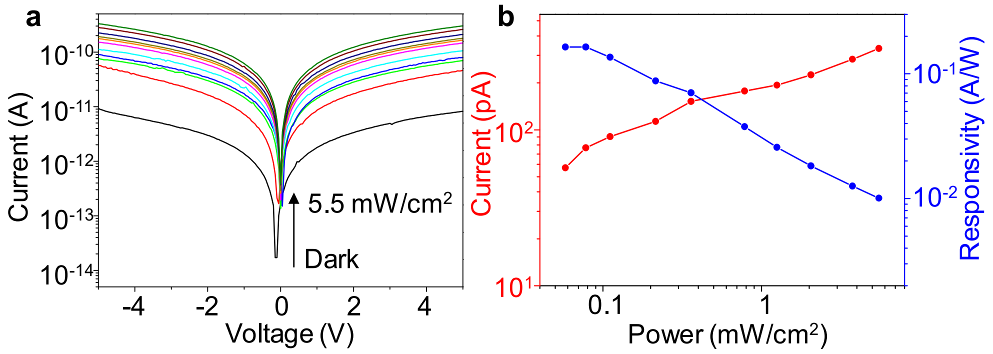

Figure S14. Polarization light response of the CHP film-based photodetector. a) Dark current and photocurrent under dark and 510 nm LCP illumination with different irradiance of the (S-MBA)$_2$PbI$_4$ film. b) Photocurrent and responsivity of the (S-MBA)$_2$PbI$_4$ film-based device under different incident power.

Figure S14a shows the (S-MBA)$_2$PbI$_4$ film detector has a representative set of the *I–V* curves under dark and 510 nm LCP illumination with different irradiance by scanning bias from -5 to 5 V. A light on/off ratio of 36.8 was calculated by extracting current value under dark and 510 nm LCP illumination at 5.5 mW/cm^2. The incident power-dependent photocurrent and responsivity of the (S-MBA)$_2$PbI$_4$ perovskite thin-film detector were plotted in Figure S14b. The highest responsivity of this film device is 0.16 A W^{-1} under irradiance of 0.06 mW/cm^2, which are nearly one order of magnitude smaller than that of the CHP array CPL photodetector, possibly because these single-crystal mircostructures have longer carrier lifetime and lower charge trap density. These results demonstrate CHP NW array devices can achieve higher photoresponse for CPL detection compared to thin-film devices.

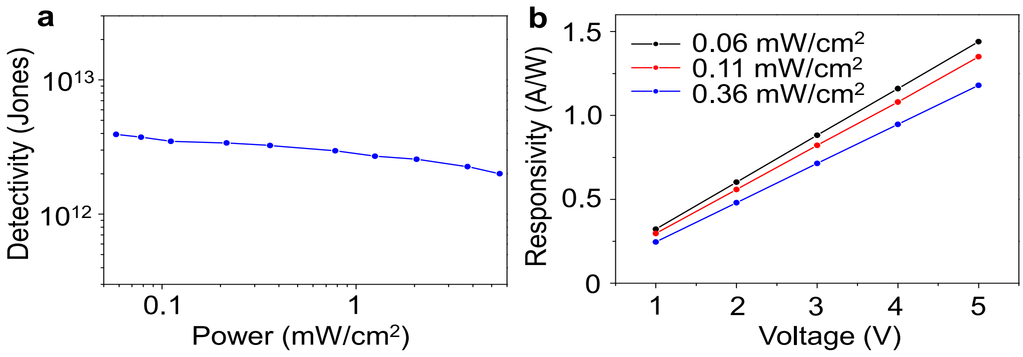

Figure S15. Detectivity and voltage-dependent performances of $(S\text{-}MBA)_2PbI_4$ NW array. a) Calculated device detectivities with different light power densities. b) Voltage-dependent responsivity of $(S\text{-}MBA)_2PbI_4$ array-based photodetector measured at various light power densities.

The detectivity (D^*) was evaluated by the formula, $D^* = \frac{I_{ph}\sqrt{S}}{P\sqrt{2eI_{dark}}}$, where e is the elementary charge. As shown in Figure S14a, the D^* decreases with increasing light intensities, reaching up to 3.9×10^{12} Jones at a low light power density of 0.06 mW/cm^2, which is higher than the reported value of chiral perovskite film photodetectors. The responsivity of $(S\text{-}MBA)_2PbI_4$ array-based photodetector also exhibits obvious voltage dependence at various light power density, which is a typical behavior of perovskite photodetectors (Figure S15b).

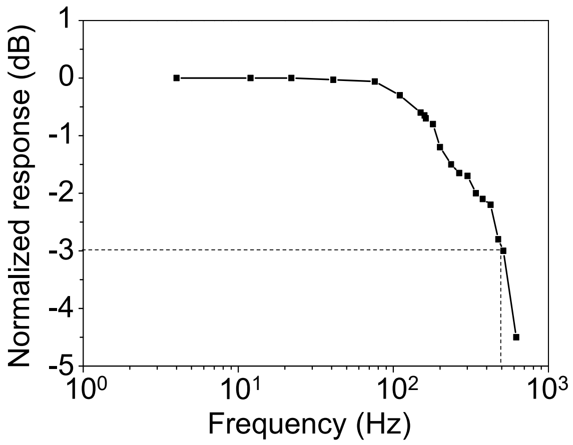

Figure S16. Normalized responses of chiral perovskite nanowire devices at various frequencies.

Figure S16 shows the normalized response of nanowire devices versus the input frequency. The -3 dB frequency, which is defined as the frequency where the response dropped to half of the initial value, was approximately 500 Hz for 510 nm response, indicating the fast response speed of single-crystal nanowire devices.

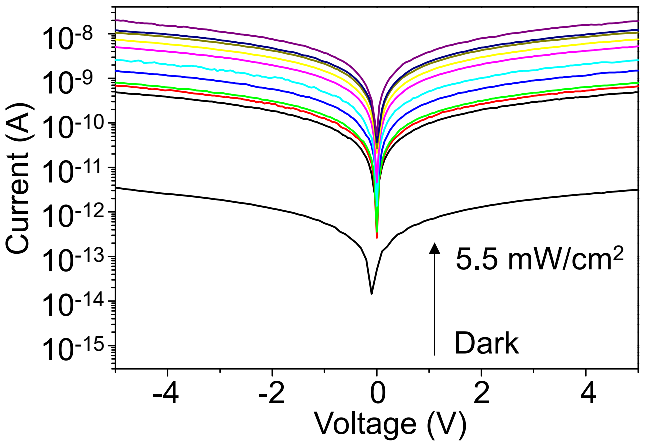

Figure S17. Current response of the $(R\text{-}MBA)_2PbI_4$ NW array under dark and RCP illumination.

As shown in Figure S17, the $(R\text{-}MBA)_2PbI_4$ single-crystalline NW array-based detector also has a representative set of the *I–V* curves under dark and 510 nm RCP illumination with different irradiance by scanning bias from -5 to 5 V. A light on/off ratio of 6.1×10^3 has been calculated by extracting current value under dark and 510 nm RCP illumination at 5.5 mW/cm^2. It can be seen that the $(R\text{-}MBA)_2PbI_4$ NW array also exhibits high photocurrent and light on/off ratio, which is in agreement with that of the $(S\text{-}MBA)_2PbI_4$ array device and validates the superior optoelectronic performance of CHP single-crystalline arrays.

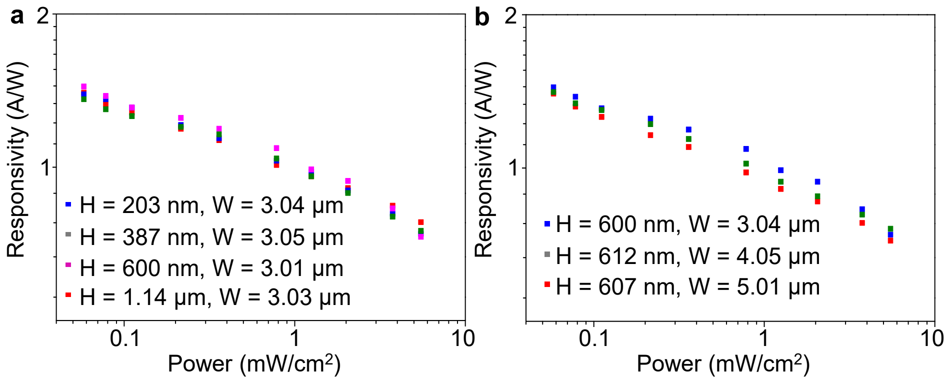

Figure S18. Responsivities of CHP NW array with different heights and widths.

As shown in Figure S18, there are no significant differences in detection performances when CHP nanowire with different sizes (widths and heights). This result demonstrates that these devices with various sizes can exhibit excellent photoresponse performance.

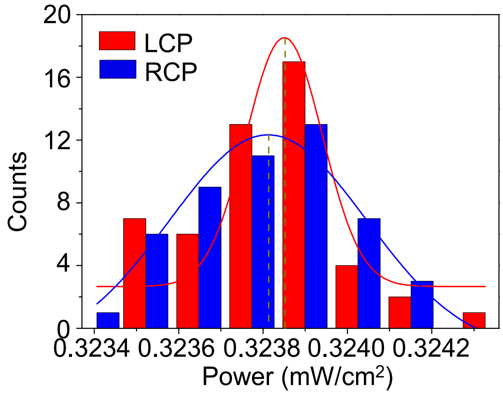

	Ave.	SD
LCP	0.3238	0.0008
RCP	0.3238	0.0007

Figure S19. The intensity calibration of LCP and RCP illumination. 100 data (50 for LCP; 50 for RCP) are counted.

Before the characterization of CPL detection performance, the whole system was calibrated to ensure that the intensity of LCP and RCP illumination was the same during all measurements.[4] Firstly, we waited 15 min to ensure the intensity of the light source (510 nm laser) was stable. Then the CPL was switched from LCP to RCP (then from RCP to LCP…) for 50 cycles, and the light intensity was monitored before each switch by a power meter. At last, 50 data were obtained for LCP and the other 50 data for RCP. As shown in the statistical graph (Fig. S19), the light intensity of LCP and RCP districted in 0.3238 ± 0.004 mW/cm^2. The relative standard deviation (defined as the ratio of the standard deviation to the mean) of LCP and RCP light intensity are 0.25% and 0.23%, which are about two orders of magnitude smaller than the $g_{I\mathrm{ph}}$ (0.1~0.3 in this paper). These results verify that the different responsibilities of LCP and RCP light are resulted from the chiral device rather than the light intensity error.

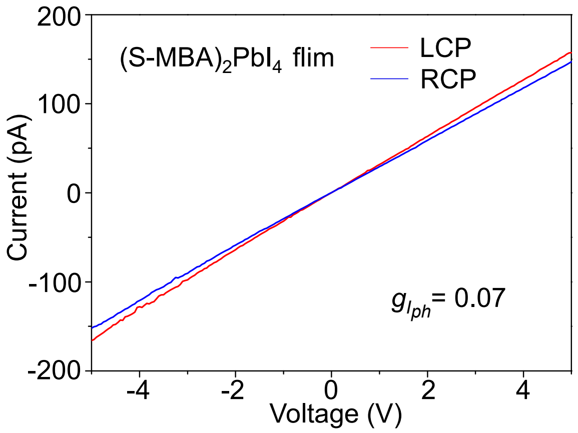

Figure S20. *I-V* curves of (S-MBA)$_2$PbI$_4$ film under 510 nm RCP and LCP illumination at the power density of 0.32 mW/cm^2.

To compare the CPL distinguishability between CHP single-crystalline NW array devices and thin-film devices, we measure the *I-V* curves under 510 nm RCP and LCP illumination for the (S-MBA)$_2$PbI$_4$ film. The $g_{I\mathrm{ph}}$ of (S-MBA)$_2$PbI$_4$ film device is calculated to be 0.07 at 5 V bias voltage, which is lower than that in the corresponding microarray device ($g_{I\mathrm{ph}}$ = 0.24, Figure 4b). The enhanced $g_{I\mathrm{ph}}$ of the CHP NW array-based detector might be attributed to long spin lifetimes originated from their excellent crystalline nature,[5-6] showing that CHP NW array devices can achieve better distinguish ability for CPL detection compared to thin-film devices.

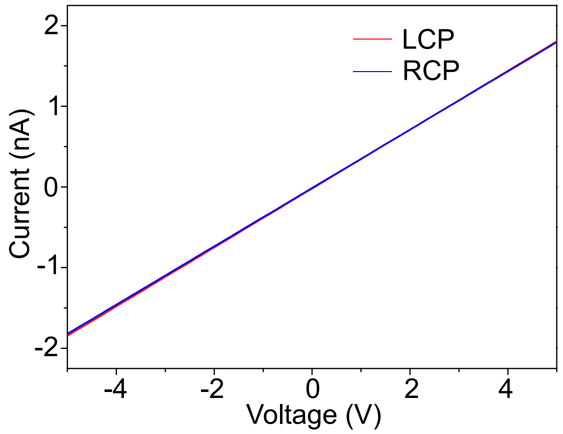

Figure S21. *I-V* curves of (rac-MBA)$_2$PbI$_4$ NW array under 510 nm RCP and LCP illumination at the power density of 0.32 mW/cm^2.

The racemic precursor (0.1 mol L^{-1}) prepared by mixing (R- and S-MBA)$_2$PbI$_4$ precursor with a molar ratio of 1:1 was used for the fabrication of (rac-MBA)$_2$PbI$_4$ NW arrays. As shown in Figure S21, the (rac-MBA)$_2$PbI$_4$ NW array-based photodetector did not show any photocurrent differences under 510 nm RCP and LCP illumination, which is significantly different from the results of (R- and S-MBA)$_2$PbI$_4$ photodetectors, indicating that the polarization distinguishability stems from the introduction of homochiral organic ligands.

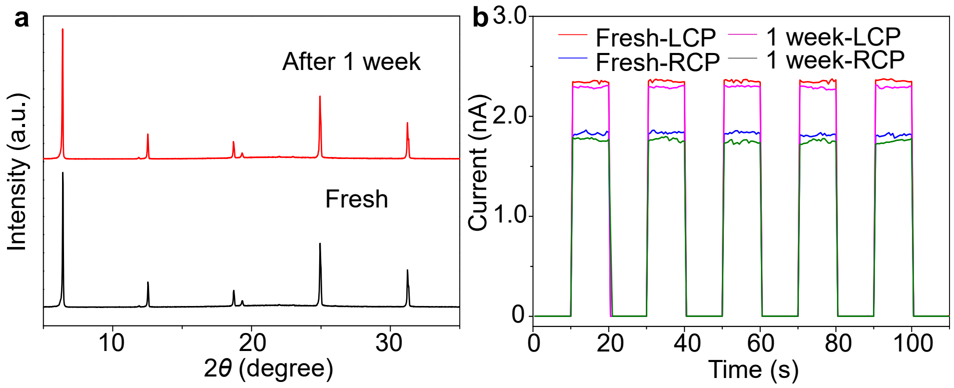

Figure S22. The ambient stability of $(S\text{-}MBA)_2PbI_4$ array. a) XRD patterns and b) Photoresponse of fresh and 1-week aged $(S\text{-}MBA)_2PbI_4$ array under LCP and RCP 510 nm light. $(S\text{-}MBA)_2PbI_4$ array are stored in a laboratory environment without any encapsulation.

The XRD pattern and photoresponse of $(S\text{-}MBA)_2PbI_4$ array were monitored after being stored in ambient without any encapsulation for 1 week to check its stability. The XRD pattern of $(S\text{-}MBA)_2PbI_4$ array revealed negligible degradation after the ambient storage, and the corresponding detector can still perform excellent polarization distinguishability, which demonstrates long-term performance of our array-based device.

References

[1] J. Ahn, E. Lee, J. Tan, W. Yang, B. Kim, and J. Moon, *Mater. Horiz.* **2017**, 4, 851.

[2] J. Feng, C. Gong, H. Gao, W. Wen, Y. Gong, X. Jiang, B. Zhang, Y. Wu, Y. Wu, H. Fu, L. Jiang, and X. Zhang, *Nat. Electron.* **2018**, 1, 404.

[3] D. Shi, V. Adinolfi, R. Comin, M. Yuan, E. Alarousu, A. Buin, Y. Chen, S. Hoogland, A. Rothenberger, K. Katsiev, Y. Losovyj, X. Zhang, P. A. Dowben, O. F. Mohammed, E. H. Sargent, and O. M. Bakr, *Science* **2015**, 347, 519.

[4] C. Chen, L. Gao, W. Gao, C. Ge, X. Du, Z. Li, Y. Yang, G. Niu, and J. Tang, *Nat. Commun.* **2019**, 10, 1927.

[5] P. Odenthal, W. Talmadge, N. Gundlach, R. Wang, C. Zhang, D. Sun, Z.-G. Yu, Z. Valy Vardeny, and Y. S. Li, *Nat. Phys.* **2017**, 13, 894.

[6] K. Liao, X. Hu, Y. Cheng, Z. Yu, Y. Xue, Y. Chen, and Q. Gong, *Adv. Opt. Mater.* **2019**, 7, 1900350.
